# *Bacillus* spp. Isolated from Miang as Potential Probiotics in Nile Tilapia Culture—In Vitro Research

**DOI:** 10.3390/microorganisms12081687

**Published:** 2024-08-16

**Authors:** Chioma Stella Anyairo, Kridsada Unban, Pairote Wongputtisin, Jiraporn Rojtinnakorn, Kalidas Shetty, Chartchai Khanongnuch

**Affiliations:** 1 Multidisciplinary and Interdisciplinary School, Chiang Mai University, Muang, Chiang Mai 50200, Thailand; chianyairo@gmail.com; 2 Division of Food Science and Technology, School of Agro-Industry, Faculty of Agro-Industry, Chiang Mai University, Muang, Chiang Mai 50100, Thailand; 3 Program in Biotechnology, Faculty of Science, Maejo University, Sansai, Chiang Mai 50290, Thailand; pairotewong@gmail.com; 4 Faculty of Fisheries and Technology, Maejo University, Sansai, Chiang Mai 50120, Thailand; jiraroj@mju.ac.th; 5 Global Institute of Food Security and International Agriculture (GIFSIA), Department of Plant Sciences, North Dakota State University, Fargo, ND 58108, USA; kalidas.shetty@ndsu.edu; 6 Department of Biology, Faculty of Science, Chiang Mai University, Huay Kaew Rd., Muang, Chiang Mai 50200, Thailand; 7 Research Center for Multidisciplinary Approaches to Miang, Science and Technology Research Institute Chiang Mai University, Muang, Chiang Mai 50200, Thailand; 8 Research Center for Microbial Diversity and Sustainable Utilization, Chiang Mai University, Huay Kaew Rd., Muang, Chiang Mai 50200, Thailand

**Keywords:** aquaculture, *Bacillus*, probiotics, Miang, Nile tilapia

## Abstract

Among 79 *Bacillus* spp. isolated from Miang, a fermented tea in north Thailand, 17 *Bacillus* strains were selected with probiotic potential in Nile tilapia culture based on the capabilities of bacteriocin production and associated antimicrobial activities against fish pathogens, *Aeromonas hydrophila* and *Streptococcus agalactiae*. However, only six isolates were selected for further extensive studies based on the strength of their antimicrobial activities and their tolerance against simulated gastrointestinal conditions. The molecular identification by 16S rRNA gene sequence analysis revealed that five isolates, K2.1, K6.1, K7.1, K15.4, and K22.6, were *Bacillus tequilensis,* and the isolate K29.2 was *Bacillus siamensis*. *B. siamensis* K29.2 showed complete susceptibility to antibiotics tested in this study, while *B. tequilensis* K 15.4 showed moderate resistance to some antibiotics; therefore, both strains were selected as potential probiotic bacteria. *B. tequilensis* K15.4 and *B. siamensis* K29.2 were capable of the production and secretion of extracellular protease and polysaccharide degrading enzymes, including cellulase, xylanase, and β-mannanase. The tannin tolerant test also demonstrated their ability to grow on selective agar plates and secrete cellulase and β-mannanase in the presence of hydrolyzable tannin. In addition, in vitro digestion of commercial fish substrate revealed that the extracellular enzymes produced by both strains efficiently reacted with feed protein and polysaccharides. Based on the results from this study, *B. siamensis* K29.2 was deemed to have the highest potential multifunctional probiotic qualities for application in Nile tilapia culture, while the antibiotic-resistant gene in *B. tequilensis* K15.4 must be clarified before field application.

## 1. Introduction

Despite its global economic and nutritional health benefits, the aquacultural sector is challenged by pathogenic microbes, which have notably resulted in diverse lethal symptoms of fish species in aquatic environments across feral, intensive, and extensive farmed conditions. Some pathogens, notably *S. agalactiae*, *A. hydrophila*, and others, top the global list of bacterial pathogens in farmed aquatic environments [1]. Although these mentioned pathogens are tagged opportunistic, they naturally inhabit the aquatic environment and are readily active throughout the year [2]. However, *S. agalactiae* is highly prevalent during the high-temperature seasons in Nile tilapia cultivation [2,3,4], while *A. hydrophila* is prevalent in low- and high-temperature freshwater bodies [5]. Both are well-established multi-host pathogens [3,6]. The lethal symptoms of their infections against fish, especially Nile tilapia (*Oreochromis niloticus*) infected by *S. agalactiae*, include irregular swimming, dorsal rigidity, lethargy, corneal opacity, exophthalmos, splenomegaly, bleeding around the eyes, operculum, fin, and body. This pathogen is revealed to be a major challenge and attacks/infects adult tilapias [2]. On the other hand, *A. hydrophila* causes an infectious disease called motile *Aeromonas* septicemia (MAS), hemorrhagic septicemia, ulcerative disease, or red sore disease, which can result in ulcerative and hemorrhagic skin ulcers [7,8,9]. Their infection is prevalent in adults and juveniles Nile tilapia [3,10]. The symptoms of both *A. hydrophila* and *S. agalactiae* have resulted in a high mortality rate of these aquatic species and economic loss for the farmers owing to their persistent reoccurring co-infection [2,3]. The use of antibiotics by farmers, amongst other adopted techniques, to address this challenge has resulted in a surge in antimicrobial resistance of the surrounding microbes. This challenge is linked to the direct and indirect disposal of aquacultural waste, lack of technical know-how on the application (especially understanding about the time frame that can allow the drug assimilation, dispersion, metabolism, and excretion, from the host fish and thus ensuring little or no presence of these drug substance in the fish before taken to the market for human and other animal consumption) and some level of failure to comply to the already established regulatory rules on antibiotics applications in fish farming [11,12,13].

Apart from the therapeutic and prophylactic applications of conventional antibiotics, some farmers also use these substances as growth promoters, thus further advancing and dispersing the drug-resistant genes of these microbes, which have yielded diverse health complications and even death in some cases across the human populace [14]. *A. hydrophila* and *S. agalactiae* are well-studied for their antimicrobial-resistant propagation and resilience via vertical gene transfer and horizontal gene transfer, respectively [15,16,17]. These multi-host pathogens are of aquacultural relevance owing to their resistance to some aquacultural approved antibiotics such as tetracycline, oxytetracycline, amoxicillin, enrofloxacin, etc. [1,9,14,18,19]. These resistances have been revealed to be due to variations in the antimicrobial resistance genes they possess, which include *erm*B, *erm*A, *erm*C, *tet*L, *tet*M, *tet*O, etc., for *S. agalactiae* and *hly*A, *act*, *exu*, *aer*A, *alt*, etc., for *A. hydrophila* [20,21]. These reoccurring resistances have placed a demand for the search for new eco-friendly antimicrobials that can abate this challenge as well as confer growth-promoting benefits to the host fish thus benefitting the farmers and ensuring the consumers’ safety [13]. For this purpose, the use of probiotics has gained global interest [22].

Probiotics are vital in aquaculture as they are ecologically friendly, pose no threat to human health, attribute nutritional and health benefits to their host fish, and serve as antimicrobials against pathogenic microbes [23]. However, studies on probiotics of *Bacillus* species are evolving due to the advantages they offer over the probiotic strains of Lactic acid bacteria (LAB), which have long been well integrated by the food, medical, industrial, and agricultural biotechnology sectors [24]. Nevertheless, the ability of *Bacillus* probiotics to have a broad spectrum of antimicrobial activity has broadened the scope of probiotics study and applications in aquaculture and other sectors [25,26,27]. In addition, an important advantage of *Bacillus* probiotics is the capability to secrete crucial enzymes such as protease, cellulase, xylanase, β-mannanase, and amylase [28,29,30,31]. These enzymes are essential in feed utilization in fishes, particularly in Nile tilapia cultivation, which is defined as an omnivorous fish that consumes both meat and plant-based feed ingredients [32]. Furthermore, Maas et al. [33] reported that the supplementation of enzymes (phytase and xylanase) and probiotics (three strains of *Bacillus amyloliquefaciens*) increased crude protein, Ca, and P digestibility in the proximal and middle gut and also microbial interactions and increased the abundance of lactic acid bacteria and *Bacillus* species. These positive effects confirmed the advantages of *Bacillus* probiotics and exogenous enzymes on nutrient availability and gut microbiome in Nile tilapia. Previous reports have proposed that potential probiotic strains of aquacultural application can be autochthonous; nevertheless, strains of non-autochthonous isolates are proven safe and efficient for application in fish farming [30,34,35,36,37,38]. In this study, our potential probiotic strains were isolated from Miang, a traditionally fermented tea leaf of *Camellia sinensis* var. *assamica,* which revealed abundances of microbial resources composed of over 80% firmicutes and 10–12% proteobacteria [39]. Some of these firmicutes *Bacillus* species were evaluated for their probiotic application in promoting human health [27]. However, there is still a study gap in revealing other advanced fields of importance wherein these *Bacillus* isolates of Miang can be of economic and public health value, especially in aquaculture, where this study proposes both growth and health impact for the potential targeted host fish species, hence the importance of this study.

This study, therefore, describes the screening for probiotic bacteria from the *Bacillus* species isolated from Miang based on their antimicrobial target against the representative of fish pathogens, *A. hydrophila* and *S. agalactiae*. The investigation of the important characteristics of probiotics for Nile tilapia culture and their beneficial properties in tolerating tannin and secreting polysaccharide and protein-degrading enzymes is also described. 

## 2. Materials and Methods

### 2.1. Bacterial Strains and Culture Condition

A total of 79 *Bacillus* isolates used for screening experiments in this study were previously isolated from Miang samples by Unban et al. [27]. All bacterial isolates were maintained in 30% (*w*/*v*) glycerol stock culture and kept at 80 °C at the Laboratory of Microbial Resources Development and Enzyme Technology, Faculty of Agro-Industry, Chiang Mai University. The *Bacillus* isolates were reactivated by culturing on nutrient agar (NA) (Himedia, Mumbai, India) under the incubation condition of 37 °C for 24 h prior to the use in all experiments. The representative fish pathogenic bacterial strains (*A. hydrophila* and *S. agalactiae*) originally isolated from infected Nile tilapia were sourced from the Faculty of Fisheries Technology and Aquatic Resources, Maejo University, Thailand. The tested pathogenic strains were cultured on Tryptic soy agar (BD, Heidelberg, Germany) at 30 °C for 24 h.

### 2.2. Screening for Bacillus Strain with Antimicrobial Activity against Fish Pathogens

A total of 79 *Bacillus* isolates from stock cultures were reactivated in 5 mL nutrient broth at 37 °C for 24 h, and all cultures were streaked on NA to confirm the strain purity and achieve a single colony. The cultures of two pathogenic bacterial strains were also prepared by inoculating their single colonies in 5 mL Tryptic soy broth (TSB) (BD, Heidelberg, Germany) and incubated under a static condition at 30 °C for 24 h. Following 24 h of culturing, the cultured broth was diluted by sterile saline 0.85 (*w*/*v*) to achieve an optical density (OD_600_) of 0.3 (approximately 10^5^ CFU/mL). The 200 µL diluted culture broth of the pathogenic bacteria was spread on NA by a sterile swab. Afterward, a single colony of *Bacillus* strains was carefully spotted against the tester microorganisms before incubating at 30 °C for 24 h [40]. A clear zone of inhibition was marked positive for inhibitory activity against the representative selected fish pathogenic bacteria, *A. hydrophila* and *S. agalactiae*.

### 2.3. Bacteriocin Producing Test by Agar-Well Diffusion Assay

The selected *Bacillus* species with antimicrobial activity against both *A. hydrophila* and *S. agalactia* were further investigated for their bacteriocin-producing capability via the agar well diffusion technique illustrated by Tagg and McGiven [41]. A single colony of the selected 29 *Bacillus* isolates was inoculated into 10 mL nutrient broth (NB) (Himedia, Mumbai, India) and incubated with agitation at 180 rpm, 37 °C, for 24 h. The vegetative culture broth was centrifuged at 12,000 rpm for 20 min at 4 °C, and the resulting cell-free culture supernatant (CFCS) was passed through a 0.22 µm pore size filter membrane (Pall Acro disc syringe filters) and aseptically kept in sterile microtube at 4 °C until use. The test strains, with a volume of 200 µL of the diluted pathogen culture, were prepared as described previously, then were transferred onto the TSA and spread using a sterile swab. Then, 6 mm diameter wells were made on the TSA using a cork borer (6 mm diameter). Afterward, 70 µL CFCS of the *Bacillus* strains neutralized with either 1 M HCl or 1 M NaOH were aseptically pipetted into the respective wells [42]. The plates were left at room temperature for 1 h for CFCS diffusion and then incubated upright at 30 °C for 24 h with sterile distilled water serving as the negative control. After the incubation, the clear inhibitory zones formed around the wells were measured according to the CLSI standard [43].

### 2.4. Acid and Bile Salt Tolerance Tests

All presumptive bacteriocin-producing isolates obtained from the previous experiment (2.3) with antimicrobial activity against fish pathogens were further investigated for their acidic and bile salt tolerance, as described previously [27]. Their cell survival rate (%) was calculated as follows: cell survival rate (%) = [final (log CFU/mL)/control (log CFU/mL)] × 100. The high survival strains were selected for further experiments to find the potential probiotic *Bacillus* strains.

### 2.5. Molecular Identification and Phylogenetic Analysis 

The genomic DNA of the six selected bacteriocin-producing *Bacillus* strains was cultivated in nutrient broth with 180 rpm agitation at 37 °C for 24 h. The bacterial cell was harvested by centrifugation at 10,000 rpm for 10 min and resuspended to achieve the appropriate volume of cell suspension for DNA extraction. Bacterial genomic DNA was extracted using a GeneJet Genomic DNA purification kit (ThermoFisher Scientific, Waltham, MA, USA) following the guidelines provided by the manufacturer. The 16S rRNA gene was amplified by polymerase chain reaction (PCR) via a MyCycler DNA thermal cycler (Bio-Rad, Hercules, CA, USA) using the genomic DNA as a template with the universal primers 27F (5′-AGA GTT TGA TCC TGG CTC AG-3′) and 1525R (5′-AAG GAG GTG WTC CAR CC-3′) as forward and reverse primers, respectively. The PCR products were purified and sequenced by the service of ATGC Co Ltd., Pathum Thani, Thailand. The nucleotide sequences of each *Bacillus* isolate were then compared and analyzed for similarities using the Basic Local Alignment Search Tools of the GenBank of NCBI. The phylogenetic and neighbor-joining analysis to construct the phylogenetic tree was performed using MEGA version 5.0 software.

### 2.6. Antibiotic Susceptibility Test of the Selected Bacillus Species

The antibiotic susceptibility test of six selected *Bacillus* isolates was tested on Mueller Hinton agar plates according to the method described by Hudzicki [43] against five selected antibiotics (erythromycin 30 µg/mL (Sigma-Aldrich, St. Louis, MA, USA), chloramphenicol 35 mg/mL (Sigma-Aldrich, MA, USA), streptomycin 20 mg/mL (MNH, Bangkok, Thailand), kanamycin 25 mg/mL (USP Biobasic, CA, USA), and ampicillin 25 mg/mL (AppliChem, DS, Darmstadt, Germany). The breakpoint was also interpreted following the Clinical and Laboratory Standards Institute (CLSI) [44].

### 2.7. Time Course Study of Growth and Antimicrobial Activity of the Selected Strains

To determine the optimal conditions for the antimicrobial activity of the two selected *Bacillus* strains against the indicator strains, a single colony of each strain was transferred into 5 mL nutrient broth (NB) and incubated at 37 °C, 180 rpm for 12 h, and the obtained culture was used as seed culture. Subsequently, 1% (*v*/*v*) of the bacterial seed culture was inoculated into fresh 100 mL NB and incubated under the same conditions for 24 h. At hourly intervals, samples were collected for enumeration of viable cells by drop plate technique on NA and incubated at 37 °C for 12 h. The viable cell count was expressed as the log value of colony-forming units per mL (log CFU/mL) [45]. Simultaneously, pH changes were also monitored at 3 h intervals using a pH meter (Ohaus starter 2100, Parsippany, NJ, USA). Samples for the analysis of optimal evaluation of antimicrobial activity were collected at 6 h intervals. The antimicrobial activity was evaluated using the agar well diffusion method, as previously described in Section 2.3. The proteinaceous nature of CFCS from the culture of both strains was also evaluated by a modified method described by Choi et al. [46]. An aliquot of the samples was co-incubated statically with an equal volume of 0.1 mg/mL Proteinase K (USB Corp, Cleveland, OH, USA) for 2 h at 37 °C. The reaction mixture was heated in a boiling water bath for 3 min, and residual antimicrobial activity was assayed against the indicator strain using the agar well diffusion method.

### 2.8. Tannin Tolerance Ability Test

To evaluate the tannin tolerance ability of the two selected *Bacillus* isolates, the agar plate screening technique of Kanpiengjai et al. [47] was modified. Briefly, NA media supplemented with 1% (*w*/*v*) CMC and LBG substrates were prepared without tannic acid (hydrolyzable tannin) and sterilized at 121 °C for 15 min. The tannin solution preparation was performed by dissolving an appropriate amount of tannin in 0.1 M sodium phosphate buffer of 7.0 pH and passing it through a 0.2 µm filter cartridge for sterilization following the method described by Kanpiengjai et al. [48]. Afterward, the tannin solution was pipetted into the molten NA at a final concentration of 1 g/L and 2.5 g/L. Another treatment that included the trypan blue-agar staining method with the same concentration of tannin (g/L) and the substrates (*w*/*v*) was also used in this study; therefore, NA plates supplemented with the substrates without tannin and NA plates supplemented with the substrates with trypan blue dye without tannin served as the control. Their tannin tolerance ability was conducted by spiking a single colony of the individual *Bacillus* isolates on these agar plates, and their growth was monitored for 48 h at 37 °C. A clear halo zone and cell growth indicated their tannin tolerance and enzyme-secreting ability.

### 2.9. Protease-Producing Capability of Selected Bacillus Strains

The submerged fermentation technique was used to assess the protease-producing ability of the selected *Bacillus* strains. Seed culture was prepared by aseptically transferring a single colony of each *Bacillus* strain into 10 mL of NB supplemented with 1% (*v*/*v*) sterile skim milk and incubated at 37 °C, 180 rpm for 24 h. The resulting seed culture was then transferred into a 250 mL Erlenmeyer flask containing 150 mL of NB-skim milk-supplemented medium. All samples were incubated in a shaker incubator at 37 °C, 180 rpm for 24 h. The time course study with 6 h intervals sampling of the culture broth was subsequently performed for 24 h, and all samples were measured for pH values using a pH meter, viable cells (log CFU/mL) by the drop plate technique on NA, and protease activity assay. The protease activity assay was as follows: inoculum sample volume of 125 µL was transferred into 125 µL 0.5% *w*/*v* casein in 10 mM phosphate buffer pH 7 and incubated at 37 °C for 10 min. This reaction was terminated with 125 µL trichloroacetic acid solution and immediately centrifuged at 8000 rpm for 10 min. Then, 250 µL of the collected sample supernatant was transferred into 1250 µL Na_2_CO_3,_ and 150 µL Folin–Ciocalteau phenol reagent was also added and vortexed. The sample mix was kept in a dark room for 30 min. Absorbance was measured at 660 nm. The enzyme assay experiments were triplicate, and the mean values and standard deviations were recorded. A standard curve for tyrosine was generated. One unit is defined as the enzyme quantity required to hydrolyze casein, resulting in a color equivalent to 1 µM of tyrosine under standard assay conditions.

### 2.10. Polysaccharide Degrading Enzymes Producing Capability of Selected Bacillus Strains

This quantitative enzyme production analysis was conducted to investigate the ability of cellulase, xylanase, and β-mannanase production of the two selected *Bacillus* species. Seed culture was prepared by transferring a single colony of the selected isolates into a 10 mL sterile NB and incubated at 37 °C, 180 rpm for 24 h. Then, 2% (*v*/*v*) of their vegetative culture was inoculated into 100 mL NB supplemented with 1% (*w*/*v*) of enzyme-inducing substrate: carboxymethyl cellulose (Sigma-Aldrich, MA, USA) for cellulase production; beechwood xylan (Megazyme, LEN, Ireland) for xylanase production; and locust bean gum (Sigma-Aldrich, MA, USA) for β-mannanase production, under the same incubation conditions. Aliquots of their samples were collected at 12 h intervals and determined for viable cells (log CFU/mL) via the drop plate technique, pH changes using a pH meter (Ohaus starter 2100, Seoul, Republic of Korea), and enzyme activity assay. The enzyme assay was performed by pelleting aliquots of the samples at 10,000 rpm, 4 °C for 10 min, and the supernatant was used for determination of the enzyme activity of cellulose, xylanase, and β-mannanase, as described by Khatthongngam et al. [49], where each enzyme activity (cellulase, β-mannanase and xylanase) was assayed using 0.5% (*w*/*v*) of the already stated substrates in triplicates for reproducibility. Glucose, mannose, and xylose were used for preparing the standard curve of reducing sugar for cellulases, β-mannanase, and xylanase, respectively. One unit of individual enzyme activity was defined as 1 µmole of reducing sugar liberated per mL of enzyme per minute.

### 2.11. In Vitro Evaluation of Polysaccharases and Proteases Activities against Fish Feed Substrate

A plant-based high protein commercial fish feed CP-9950 (Charoen Pokphand Foods Public Co., Ltd., Bangkok, Thailand) with an average size of 3 mm was used as a substrate for in vitro digestibility test of polysaccharases and proteases produced by the selected strain. A weight of 10 g fish feed was transferred into 100 mL of 50 mM sterile sodium phosphate buffer pH 7.0 supplemented with chloramphenicol to achieve the final concentration of 35 mg/mL to inhibit bacterial growth. Then, 1 mL of crude protease obtained from the previous experiment (Section 2.9) was added, mixed well, and incubated at 30 °C with gentle shaking at 100 rpm to ensure the enzyme reaction homogeneity. The reaction mixture was sampled at a 1 h interval from 0 to 8 h, and the collected samples were centrifuged at 8000 rpm at 4 °C The supernatant was determined for free alpha amino acid content, released protein, and protein concentration using the ninhydrin method and Bradford assay, respectively [50,51]. In the case of the proteolytic activity test, crude polysaccharases (an equal volume of their produced crude β-mannanase, xylanase, and cellulase) obtained from the previous experiment (Section 2.10) were mixed to react with the commercial fish feed CP-9950 similarly to the case of protease and incubated with the same condition. The reaction mixture was sampled and analyzed for reducing sugar by the DNS method described previously. The sampled fish feed without the enzyme addition served as the control. All analysis was measured in triplicates, and the resulting mean values and standard deviation were ascertained statistically.

### 2.12. Statistical Analysis

The experiments were conducted in triplicate, and the results are presented as mean values ± standard deviations (SD) in both tables and figures. To assess significant differences between the samples, a one-way analysis of variance (ANOVA) was employed, with a significance level set at *p* < 0.05. Data analysis was performed using SPSS 26.0 (SPSS Inc., Chicago, IL, USA), and graphical representations were generated using OriginPro 2022 (version 9.9, MicroCal Software, Northampton, MA, USA).

## 3. Results and Discussion

### 3.1. Screening of Anti-Fish Pathogen Bacillus Strain by Soft Agar Overlay Technique

**Figure 1:**
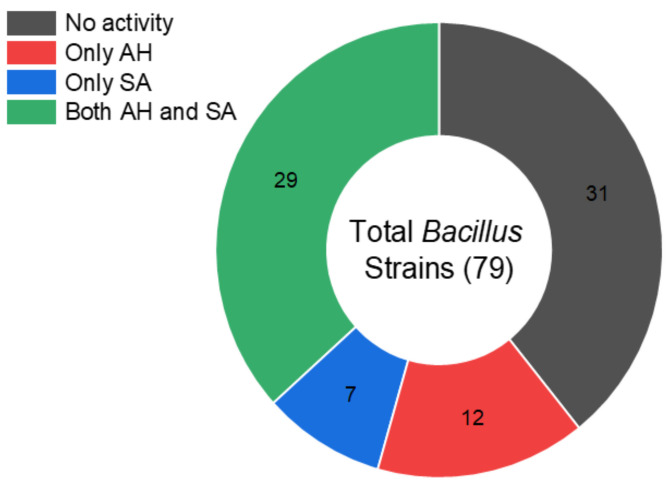
Pie chart illustrating the total number of screened *Bacillus* strains and their inhibitory activities; (no activity) are strains with no inhibitory activities against the indicator strains; (only AH) are those that inhibited only *A. hydrophila*; (only SA) are those that inhibited only *S. agalactiae*; (both AH and SA) inhibited both indicator strains.

Among the 79 isolates, 29 *Bacillus* species showed antimicrobial activity against *A. hydrophila* and *S. agalactiae* by the soft agar overlay technique (Figure 1), indicating their ability to inhibit these Gram-positive and Gram-negative pathogenic microbes, which is one of the presumptive properties of *Bacillus* species for potential as probiotics [52,53].

### 3.2. Bacteriocin-Producing Test by Agar-Well Diffusion Assay 

All 29 selected *Bacillus* species that were confirmed from the soft agar overlay screening were further subjected to an agar well diffusion assay to reconfirm their antimicrobial potential against the test strains according to the CLSI standard. The results revealed that only 17 strains, as shown in Table 1, reproduced their antimicrobial activities against the test strains used in this assay, with the *Bacillus* sp. K19.1 showed the highest inhibitory activity (21.8 ± 0.28 mm) against the important pathogen *S. agalactiae* followed by the isolate K14.4 (21.5 ± 0.71 mm), K15.4 (20.5 ± 0.14 mm), and K7.1 (20.3 ± 0.35), with the lowest inhibitory activity found in the isolate K10.4 (6.8 ± 0.35 mm). For *A. hydrophila*, the isolates K27.2 and K29.2 showed the highest inhibitory activities (19.6 ± 0.50 mm and 19.1 ± 0.28 mm), followed by the isolate 7.1 (19.1 ± 0.71 mm), while the isolate K13.3 (6.6 ± 0.57 mm) had the lowest inhibitory activity. However, the remaining twelve *Bacillus* isolates, K1.3, K3.3, K4.2, K6.2, K14.1, K19.3, K21.1, K25.1, K25.2, K25.4, K26.1 and K31.3, had no inhibitory zone. The utilization of the agar well diffusion technique for this study is due to its distinct advantage of allowing the produced bacteriocin to diffuse into the agar medium before the tester microbes start propagating, thus ensuring an enhanced sensitivity of the tester strain around the inoculated well area leading to improved antimicrobial activity, and again it provides a well-defined comparison platform for testing the bacteriocin-producing ability of different strains under the same condition [41].

### 3.3. Acid and Bile Salt Tolerances Test

As per the recommendations of the FAO/WHO, it is imperative for potential probiotic strains to exhibit resilience against the harsh acidic conditions in the gastrointestinal tract [22]. This resilience ensures their effectiveness in achieving the desired results for high-throughput applications. It is worth noting that the bile concentration in the gastrointestinal tract of Nile tilapia can vary due to their diet composition, and their physiological bile concentration ranges from 0.4 to 1.3%, with the gastric pH from 1 to 7 [54,55,56,57]. In light of this, all 17 selected *Bacillus* species were subjected to an assay under conditions of 0.3% (*w*/*v*) bile salt and acidic pH (pH 2 and 3) for 3 h. The results presented in Table 2 revealed that only six *Bacillus* species (K2.1, K6.1, K7.1, K15.4, K22.6, and K29.2) displayed increased resistance under these harsh conditions. 

Their survival rates (%) in the presence of bile salt ranged from 86 to 99%, and K15.4 and K29.2 had the highest survival rates at 99 and 98%, respectively. All tested strains showed a variety of tolerance against acidic conditions at pH 2 and pH 3. Among these strains, the isolates K29.2 and K15.4 showed the highest survival rate at 73 and 65% at pH 2, respectively, while the lowest survival rate was found with isolate K14.4 at only 19%. The survival rates of all tested strains against acidic conditions at pH 3 were found to correspond with those of pH 2, in which the isolates K29.2 and K15.4 were also the highest compared to others. Based on the resistance to the simulated conditions of the gastrointestinal tract of Nile tilapia mentioned previously and the ability of antimicrobial activity against *A. hydrophila* and *S. agalactiae* (Table 1), the *Bacillus* spp. K2.1, K6.1, K7.1, K15.4, K22.6, and K29.2 were selected for further studies. 

**Figure 2:**
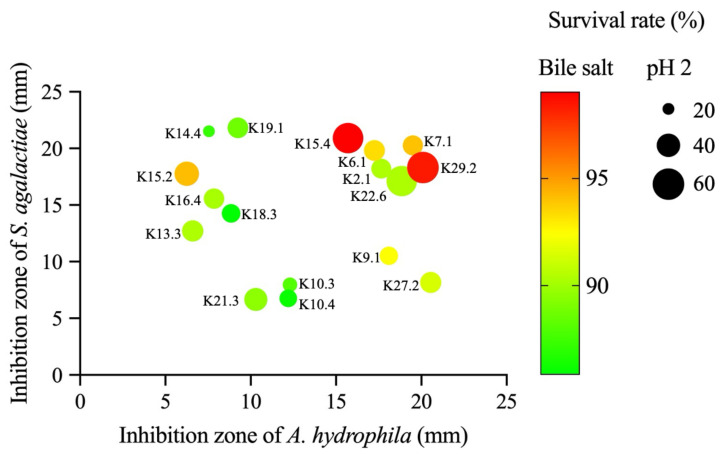
The relationships of antimicrobial activities against two pathogens, *S. agalactiae* and *A. hydrophila*, in comparison to the survival rate against bile salt and acidic conditions of the selected *Bacillus* strains. Differences in color and size of circulars represent the degree of survival against bile salt and acidic conditions.

Based on the results of antimicrobial activities of the selected 17 bacteriocin-producing *Bacillus* spp. (Table 1) and their survival rate against the simulated gastrointestinal conditions, 0.3% (*w*/*v*) bile salts and acidic conditions; the comparative diagram demonstrating the relationship of those characteristics is presented in Figure 2.

### 3.4. Bacterial Identification and Phylogenetic Relationships

Molecular identification is a widely accepted technique for ascertaining even the smallest differences in the nucleotide sequences of microbes within the same genus. This technique is highly reproducible and less labor-intensive [58]. In our study, all six potential probiotic *Bacillus* spp. Were selectively identified based on the 16S rRNA gene sequence analysis, and it was found that five *Bacillus* isolates, K2.1, K6.1, K7.1, K15.4 and K22.6, were identified to be *B. tequilensis* with a close 99–100% similarity to the type strains (Table 3), while the isolate K29.2 was revealed to be *B. siamensis* with 99.93% similarity to the type strain.

**Figure 3:**
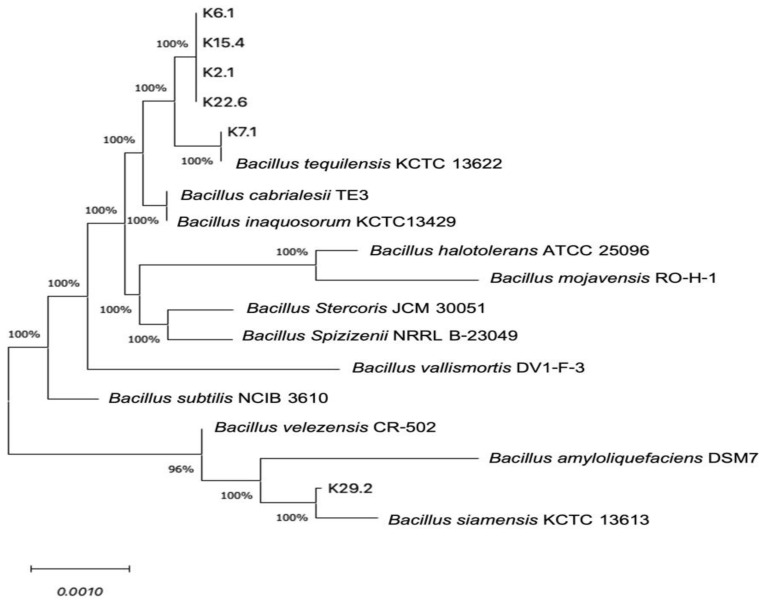
The phylogenetic tree of selected *Bacillus* species constructed by the neighbor-joining method based on 16S rDNA gene sequence analysis. Bootstrap values > 50% (based on 1000 replications) are given at branch points. The scale bar shows a patristic distance of 0.0010.

The phylogenetic analysis, based on the 16S rDNA nucleotide sequences of these isolates, was compared with other known *Bacillus* sequences available in the GenBank database using the neighbor-joining comparison method. The results, depicted in Figure 3, showed their close similarity to *B. subtilis* of the GRAS category of FAO/WHO [22]. This closeness agrees with previous studies [59,60].

### 3.5. Antibiotic Susceptibility Profile of the Potential Probiotic Bacillus Species

The safety of potential probiotic strains is paramount as there can be a possibility of cross or horizontal gene transfer in terms of antibiotic resistance [13,61]. To this effect, our study considered these *Bacillus* safety by their antibiotic susceptibility evaluation against some selected classes of antibiotics: the macrolides (erythromycin and chloramphenicol), the aminoglycosides (streptomycin and kanamycin), and the beta-lactam (ampicillin) according to CLSI standard as illustrated by Hudzicki [43]. Referencing our results, as shown in Table 4, to the CLSI standard antibiotics breaking points [44], *B. siamensis* K29.2, *B. tequilensis* K2.1, and *B. tequilensis* K22.6 exhibited susceptibility to all classes of antibiotics selected for this study. However, *B. tequilensis* K7.1 displayed resistance to the two aminoglycosides (streptomycin and kanamycin), while *B. tequilensis* K15.4 and K6.1 were resistant only to streptomycin. Notably, only *B. tequilensis* K15.4 demonstrated resistance to the beta-lactam antibiotic ampicillin.

It is important to mention that a similar resistance pattern has been reported previously [62]. Furthermore, a study by Gueimonde et al. [63] noted that antibiotic resistance in probiotic bacteria, resulting from mutations or intrinsic resistance mechanisms, is non-transferable. This provides an additional advantage to these strains as they can contribute to the restoration of their host’s gut microbiota after exposure to antibiotics. Moreover, the antibiotic resistance observed in *B. tequilensis* is confirmed to be intrinsic, further ensuring their safety for probiotic applications [62,64]. However, the presence of the transferable antibiotic resistance gene of *B. tequilensis* K15.4 must be scientifically confirmed before practical application. According to the characteristics of both the antimicrobial activity against fish pathogenic bacteria and the resistance to bile salt and acidic conditions, *B. siamensis* K29.2 has the highest potential as multifunctional probiotic bacteria for application in Nile tilapia culture, while the antibiotic-resistant gene in *B. tequilensis* K15.4 has to be clarified before field application. 

### 3.6. Time Course of Growth and Antimicrobial Activity of the Selected Strains

**Figure 4:**
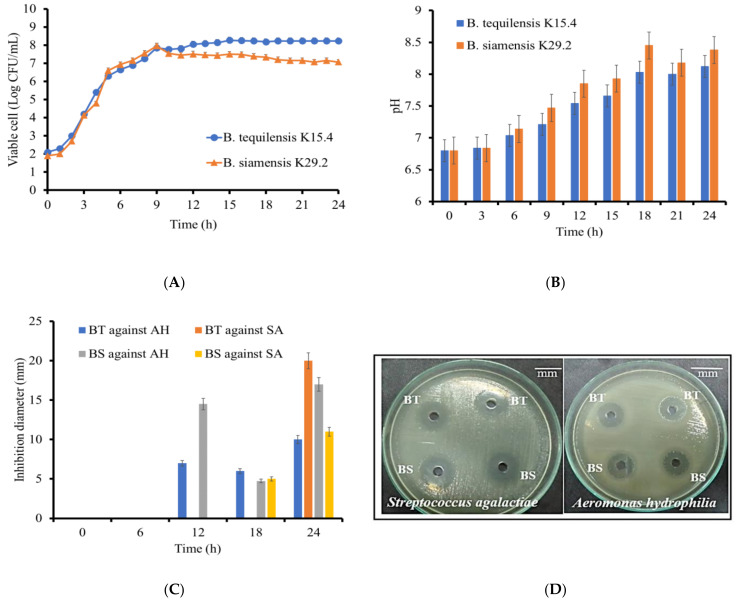
Characteristic growth dynamics of *B. tequilensis* K15.4 and *B. siamensis* K29.2 and their antimicrobial activities: (**A**) line graph representing the growth curve of the selected *Bacillus* species; (**B**) pH changes; (**C**) inhibitory activity (mm) of the CFCS collected at 6 h interval against the test strains; (**D**) inhibitory activity via agar well diffusion of 24 h collected CFCS against the test strains. Data expressed as mean ± SD (N = 3). BT = *B. tequilensis* K15.4; BS = *B. siamensis* K29.2; SA = *S. agalactiae*; AH = *A. hydrophila.*

Understanding the growth dynamics of a microbial strain is crucial for optimizing its performance and productivity. This study aimed to establish the optimal conditions for bacteriocin production in *B. tequilensis* K15.4 and *B. siamensis* K29.2 under the chosen cell proliferation conditions. The 24 h time course study of individual *Bacillus* species growth curves, as depicted in Figure 4A, revealed that the exponential growth phase from the initial stage to 9 h of *B. tequilensis* K15.4 was almost similar to that of *B. siamensis* K29.2. However, the overall maximum viable cell number of *B. tequilensis* K15.4 after 9 h cultivation was higher than that of *B. siamensis* K29.2. The pH changes during the bacterial growth (Figure 4B) indicated that both strains maintained a consistently basic profile, up to pH 8.3 for *B. siamensis* K29.2 and pH 8.12 for *B. tequilensis* K15.4, highlighting their alkaline pH nature. Comparisons with other studies culturing *B. siamensis* and *B. tequilensis* in media containing MgSO_4_.7H_2_O, like MRS [46,65], suggest that the final culture pH is influenced by the composition of the culture media.

The antimicrobial activity of the collected cell-free culture supernatants (CFCS) at 6 h intervals revealed significant variations over incubation times. The statistical analysis in Figure 4C displayed the in vitro antimicrobial activity progression for both strains at 0, 6, 12, and 24 h, testing against the indicator strains (*A. hydrophila* and *S. agalactiae*). No inhibition against *A. hydrophila* was observed from 0 to 6 h CFCS, but at 12 h, *B. siamensis* (14.5 ± 1.0 mm) and *B. tequilensis* (7 ± 1.0 mm) exhibited inhibitory activity. Although the 18 h bacteriocin activity of *B. tequilensis* (6 ± 0.5 mm) outperformed that of *B. siamensis* (4.75 ± 0.9 mm), the 24 h activity increased significantly (10 ± 0.8 mm) and (17 ± 0.7 mm), as shown in Figure 4D. However, the sharp reduction in antimicrobial activity at 18 h is hypothesized to result from rapid physiological and biochemical changes during the initial stationary growth phase, as indicated in Figure 4A. The conditions later recovered potentially through secondary metabolism, consistent with the typical stationary phase of bacterial growth [66].

Moreover, in the timed CFCS inhibition against *S. agalactiae*, only the 18 h *B. siamensis* showed activity (5 ± 0.7 mm) in 0–18 h exposure to CFCS. However, their 24 h CFCS response demonstrated inhibition of (20 ± 0.5 mm) for *B. tequilensis* and (11 ± 0.4 mm) for *B. siamensis*, aligning with their initial screening results.

The effect of proteinase K activity on the antimicrobial potency of the individual CFCS revealed a residual antimicrobial activity (%) of *B*. *tequilensis* K15.4 to be 55.6 ± 0.67 and that of *B*. *siamensis* K29.2 to be 80 ± 0.86. This activity reduction reconfirms their proteinaceous nature, as this response is characteristic of bacteriocins as proteins [67]. However, the bacteriocins in CFCS from our selected strains showed resistance to the activity of protease K used in this experiment, especially *B*. *siamensis* K29.2 bacteriocin. Some previous reports described the resistance of bacteriocins against some proteolytic enzymes [68,69]. Nevertheless, these properties require further investigation after these bacteriocins have been purified.

### 3.7. Proteolytic Activity of the Bacillus Species

**Figure 5:**
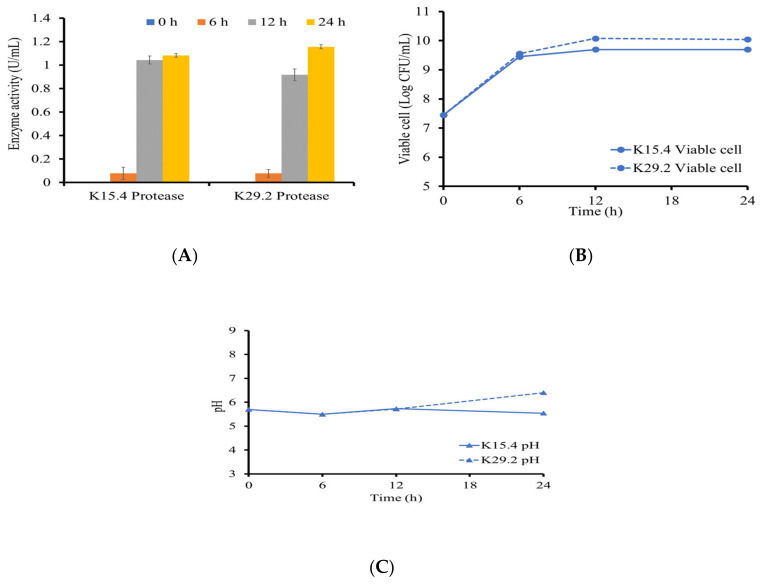
Protease production by *B. tequilensis* K15.4 and *B. siamensis* K29.2: (**A**) viable cell (log CFU/mL); (**B**) pH changes; (**C**) enzyme activities (U/mL). Data expressed as mean ± SD (N = 3).

**Figure 6:**
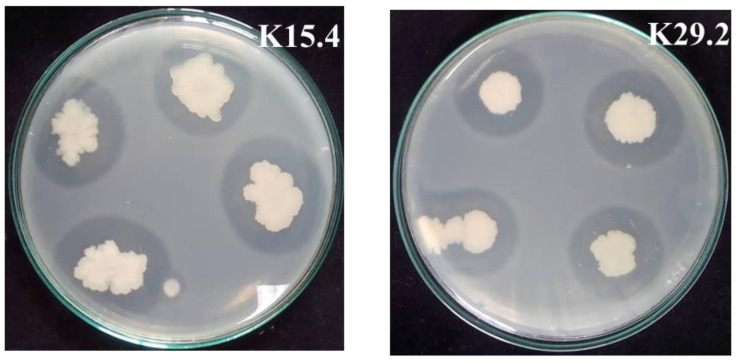
Qualitative proteolytic activity of the selected *Bacillus* strains on NA supplemented with 10 g/L skim milk indicating their clear zone of hydrolysis catalyzed by extracellular protease activity.

The selected *Bacillus* species from this study demonstrated the ability to synthesize and secrete the proteolytic enzymes extracellularly, as depicted in Figure 5 and Figure 6. Both strains have their highest activity at 24 h, with *B. siamensis* K29.2 having an activity of 1.156 ± 0.854 U/mL and 1.082 ± 0.089 U/mL for *B. tequilensis* K15.4 (Figure 5C). The proteolytic activities of both *B. tequilensis* K15.4 and *B. siamensis* K29.2 were detected after 6 h cultivation and showed the highest activity at 24 h. However, the difference in proteolytic production between the selected two species is that the enzyme activity of *B. tequilensis* K15.4 was highly detected at 12 h cultivation, which is not significantly different from the maximized activity at 24 h. The pH change study also revealed their alkaline nature, especially *B. siamensis* K29.2 (Figure 5B). Since the aim of this study was to ascertain the protease production ability of *B. tequilensis* K15.4 and *B. siamensis* K29.2, these results confirmed the capability of both strains in the production of proteolytic enzymes. In addition, their ability to grow and secrete protease in a cost-effective medium also revealed the industrial potential of these strains, particularly *B. siamensis* K29.2. The importance of the proteolytic activity of potential probiotic strains in the aquacultural sector cannot be overemphasized [70]. Over the years, protease utilization as a fish feed additive has increased due to the increasingly high levels of indigestible proteins in some cereals and grains used in fish feed formulation, and this enzyme is essential for their protein metabolism, enabling feed utilization [70,71]. In Nile tilapia, the protease activity of their gastrointestinal tract and its surrounding tissues are well-established [54]. However, studies have shown that the application of *Bacillus* probiotic strains enhances this digestive enzyme activity, resulting in gut microbial modification and improved feed digestibility by the increasing abundance of these beneficial microbes [38].

### 3.8. Polysaccharide Degrading Enzyme Production of the Selected Bacillus Species

**Figure 7:**
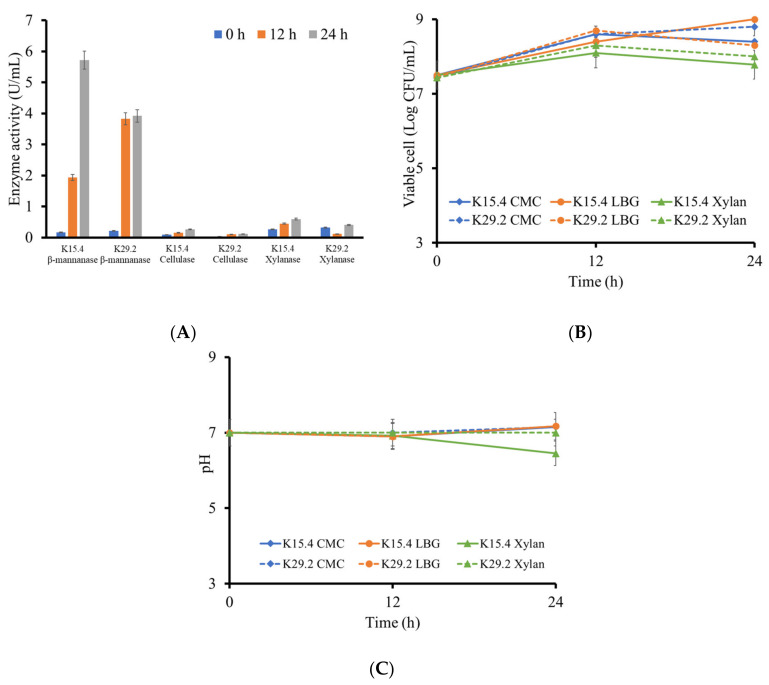
The quantitative analysis of the polysaccharide degrading enzyme (β-mannanase, cellulase, and xylanase) of *B. tequilensis* K15.4 and *B. siamensis* K29.2: (**A**) enzyme activities (U/mL); (**B**) the viable cell (log CFU/mL); (**C**) pH changes. Data expressed as mean ± SD (N = 3).

The application of probiotic *Bacillus* species with the ability to secrete plant cell wall polysaccharides degrading enzymes, amongst other effective probiotic properties as a fish feed additive or water supplement, is proven to improve fish feeding appetite, growth performance, feed utilization, flesh quality, and reduced malformations [23,26,72,73]. For this purpose, we quantitatively assayed the ability of *B. tequilensis* K15.4 and *B. siamensis* K29.2 to secrete cellulase, xylanase, and β-mannanase enzymes across different time intervals within 24 h. This study found that there was a progressive increase in cellulase activity (U/mL) across the different incubation time intervals, even though this activity is low compared to the other evaluated enzyme activities of this study (Figure 7A). However, this cellulase activity range is obtainable with most cellulase-secreting *Bacillus* species except for those assessed under diverse optimized conditions [74,75,76,77]. *B. tequilensis* K15.4 produced higher cellulase activity than *B. siamensis* K29.2. The viable cells of *B. tequilensis* K15.4 maintained a steady increase from 7.50 ± 0.03 at 0 h to 8.40 ± 0.10 (log CFU/mL) at 12 h, but *B. siamensis* K29.2 increased steadily from 7.45 ± 0.20 at 0 h to 8.60 ± 0.10 at 12 h and 8.80 ± 0.09 (log CFU/mL) at 24 h (Figure 7B). In addition, the pH changes in the culture from both strains were almost the same throughout the cultivation time (Figure 7C). The β-mannanase production was found to be higher than the other evaluated enzymes, as presented in Figure 7A. This result shows that the 24 h *B. tequilensis* K15.4 β-mannanase activity (5.72 ± 0.10 U/mL) was higher than that of *B. siamensis* K29.2 (3.92 ± 0.03 U/mL). The cell viability for *B. tequilensis* K15.4 maintained an increasing trend (7.52 ± 0.01 at 0 h, 8.41 ± 0.01 at 12 h, and 9 ± 0.01 log CFU/mL at 24 h). *B. siamensis* K29.2 fluctuated between 12 and 24 h (7.45 ± 0.09 at 0 h, 8.7 ± 0.01 at 12 h, and 8.3 ± 0.2 log CFU/mL at 24 h) (Figure 7B). Their pH across the study time varied between 7 and 8 (Figure 7C). This outcome corresponds to the results reported by Khatthongngam et al. [48]. The two strains also showed the ability to secrete xylanase across the different time courses. This study outcome is similar to the *Bacillus* strain reported by Yopi et al. [78].

The overall results from this experiment confirm the capability of *B. tequilensis* K15.4 and *B. siamensis* K29.2 to produce polysaccharide degrading enzymes, which are possibly beneficial to assist in the utilization of nutrients in fish feed. Nile tilapia naturally exhibits the omnivorous feeding mode; nevertheless, it is a high-plant-food-consuming fish [79]. This feeding mode is often limited in intensive farming care, wherein their nutritional need is supplemented with plant sources such as rice bran, raw soybean meal, wheat bran, rape seed meal, cotton seed, and leaf meal, among various options. These feed additives are often associated with some antinutritional factor (ANF) such as protease inhibitors, tannins, non-starch polysaccharides, oligosaccharides and antivitamins, phytates [80,81,82]. The findings from this research strongly support the possibility of applying *B. tequilensis* K15.4 and *B. siamensis* K29.2 as probiotics in the commercial farming of omnivorous fish such as Nile tilapia.

### 3.9. Tannin Tolerance Determination

**Figure 8:**
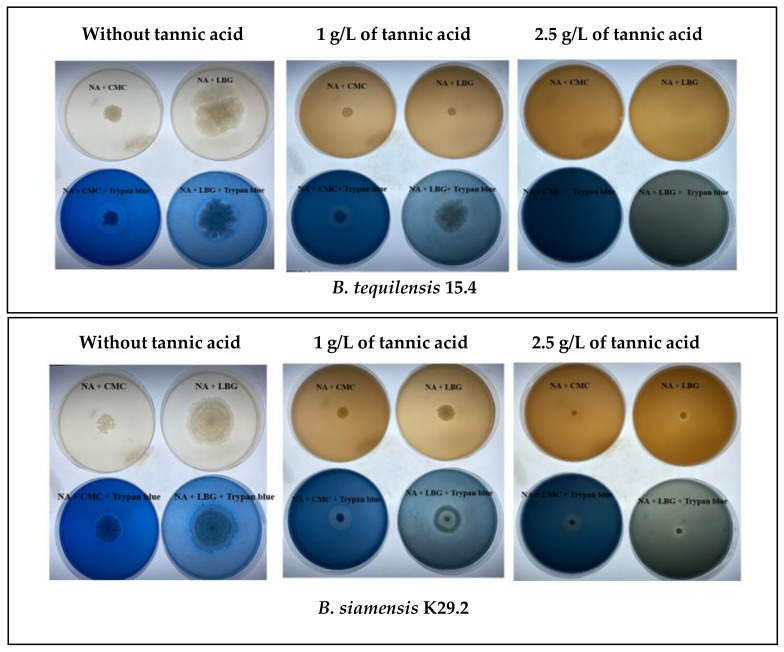
Tannin tolerant ability of *B. tequilensis* K15.4 and *B. siamensis* K29.2 at different concentrations of tannic acids (1 and 2.5 g/L) with enzyme substrate CMC (carboxymethyl cellulose) and LBG (locust bean gum), without and with trypan blue at 37 °C for 48 h. The red arrow indicates the hydrolysis zone.

Tannin is an antinutritional factor (ANF) associated with plant-based fish feed supplementation [83]. Studies have attributed some beneficial effects to tannin feed supplementation, such as lipid peroxidation reduction in the muscle of tilapia and antimicrobial effects [84,85]. The inclusion of raw plant-based ingredients in fish feed is of great concern due to the negative effects of dietary tannins, such as inhibition of digestive enzymes like protease, amylases, and lipases, resulting in the formation of indigestible complexes, poor feed utilization, reduced feed intake, and ultimately diminished the growth performance [86,87,88]. This study evaluated in vitro the ability of the selected *B. tequilensis* K15.4 and *B. siamensis* K29.2 to tolerate and produce important polysaccharide degrading enzymes in the presence of 1 and 2.5 g/L tannic acid. The result revealed that both selected *Bacillus* species can effectively grow, degrade tannin, and secrete extracellular cellulase and β-mannanase at 1 g/L tannic acid NA supplemented with these enzyme substrates CMC and LBG (Figure 8). However, at a higher concentration of tannic acid (2.5 g/L), only *B. siamensis* K29.2 was able to maintain these activities, while the growth of *B. tequilensis* K15.4 was inhibited. The tannin tolerance ability of *B. siamensis* and *B. tequilensis* has been reported [27,48]. However, to the best of our knowledge, this is the first study to evaluate the ability of *B. tequilensis* K15.4 and *B. siamensis* K29.2 to produce these important digestive enzymes in the presence of tannin with regard to their potential application as probiotics in Nile tilapia cultivation. Hence, there is a need for further in vivo study to ascertain their probiotic effects towards moderating the impact of tannin present in plant-based supplemented fish feed in the potential host Nile tilapia, revealing the possible influence of these digestive enzymes in the Nile tilapia gastrointestinal tract. 

### 3.10. Evaluation of Polysaccharases and Proteases Activities on Feed Polysaccharides and Proteins

**Figure 9:**
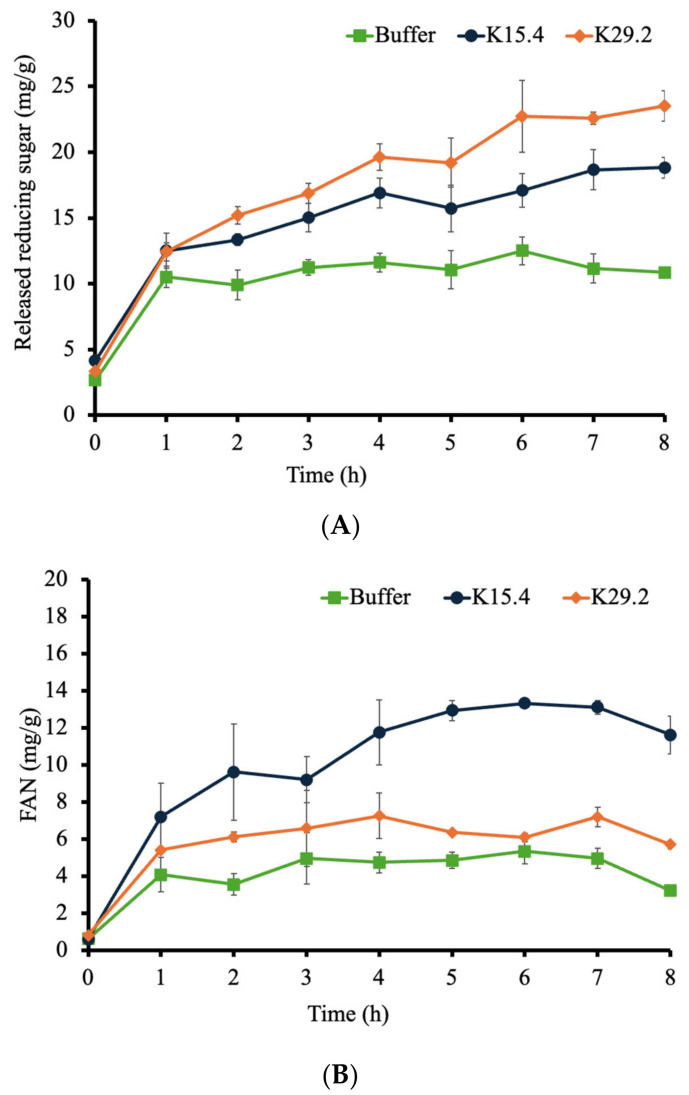
Digestion of commercial fish feed by crude polysaccharases and proteases from *B. tequilensis* K15.4 and *B. siamensis* K29.2: (**A**) released reducing sugar (mg/g) by polysaccharase; (**B**) released free alpha-amino acid (FAN) (mg/g) by crude protease.

This in vitro study revealed that the produced polysaccharide degrading enzymes of *B. tequilensis* K15.4 and *B. siamensis* K29.2 could react with polysaccharides in the commercial fish feed. Applying these crude enzymes in the reaction mixer yielded an increase in the released reducing sugar (mg/g) and free alpha-amino acid (FAN) (mg/g) (Figure 9). As this study aimed to advance the potential *Bacillus* spp. for use as a probiotic and apply as a feed additive in Nile tilapia culture, the result from this experiment confirmed the additional nutritional benefits of the *B. tequilensis* K15.4 and *B. siamensis* K29.2. Due to the presence of antinutrients such as protease inhibitors, tannins, non-starch polysaccharides, oligosaccharides, antivitamins, and phytates in plant-based-protein fish feed, the impact of the drawback effect of these antinutritional factors is essentially required [80,81,82,89]. The application of *B. tequilensis* K15.4 and *B. siamensis* K29.2 as fish feed addictive would serve as an effective strategy to mitigate the potential adverse impacts arising from the presence of antinutrients and high released protein content challenges in fish farming, thereby enhancing the nutritional quality of plant and high protein-based ingredients. 

## 4. Conclusions

This study elucidated the screening of probiotic potentials of the 79 *Bacillus* species isolated from fermented tea Miang for application in Nile tilapia cultivation. Of these strains, 17 isolates demonstrated consistent inhibition against both tested fish pathogenic bacteria *A. hydrophila* and *S. agalactiae*. Among these, six *Bacillus* isolates were selected according to their high antimicrobial activities against both pathogens and the tolerances to bile acid and stimulated gastric conditions. Five isolates were identified to be *B. tequilensis* K2.1, *B. tequilensis* K6.1, *B. tequilensis* K7.1, *B. tequilensis* K15.4, and *B. tequilensis* K22.6, and the remaining isolate was *B. siamensis* K29.2 through the molecular identification by 16S RNA gene sequence analysis. Furthermore, the result from the antibiotic susceptibility test indicated that only two strains, *B. tequilensis* K15.4 and *B. siamensis* K29.2, show their safety as probiotic strains. These selected *Bacillus* spp. are protease and polysaccharase producers and secreted those beneficial enzymes as extracellular enzymes. These results implied their multifunctionality as efficient producers of antimicrobials against important fish pathogenic bacteria and as potential growth promoter probiotics in Nile tilapia culture. This valuable application would be helpful in reducing the use of antibiotics and promoting sustainable aquaculture. Nevertheless, further studies on the identification and characterization of bacteriocins produced by *B. tequilensis* K15.4 and *B. siamensis* K29.2 are of interest for more effective uses as either prophylactic or therapeutic antimicrobials in Nile tilapia culture. In addition, the idea of combining the selected *Bacillus* strains with Miang tea or its extract to formulate the synbiotic product has also gained interest.

## Figures and Tables

**Table 1:** Confirmatory antimicrobial activity of the selected *Bacillus* strains via agar-well diffusion assay.

*Bacillus* Strains	Antimicrobial Zone of Inhibition (mm)	
	*A. Hydrophila*	*S. Agalactiae*
K2.1	17.7 ± 0.21 ^d^	18.2 ± 0.28 ^d^
K6.1	17.3 ± 0.35 ^d^	19.8 ± 0.28 ^c^
K7.1	19.5 ± 0.71 ^b^	20.3 ± 0.35 ^bc^
K9.1	18.1 ± 0.14 ^cd^	10.5 ± 0.71 ^h^
K10.3	12.3 ± 0.28 ^f^	8.0 ± 0.10 ^i^
K10.4	12.2 ± 0.28 ^f^	6.8 ± 0.35 ^j^
K13.3	6.6 ± 0.57 ^k^	12.7 ± 0.42 ^g^
K14.4	7.6 ± 0.07 ^j^	21.5 ± 0.71 ^ab^
K15.2	6.3 ± 0.35 ^k^	17.8 ± 0.35 ^d^
K15.4	15.7 ± 0.28 ^e^	20.9 ± 0.14 ^abc^
K16.4	7.9 ± 0.50 ^ij^	15.6 ± 0.64 ^e^
K18.3	8.9 ± 0.21 ^hi^	14.3 ± 0.35 ^f^
K19.1	9.3 ± 0.35 ^gh^	21.8 ± 0.28 ^a^
K21.3	10.3 ± 0.42 ^g^	6.7 ± 0.50 ^j^
K22.6	18.9 ± 0.21 ^bc^	17.1 ± 0.42 ^d^
K27.2	20.6 ± 0.50 ^a^	8.2 ± 0.21 ^i^
K29.2	20.1 ± 0.28 ^bc^	18.3 ± 0.42 ^d^

Note: Means in columns with different superscripts are statistically different at *p* < 0.05.

**Table 2:** Acidic conditions and bile tolerance test of the *Bacillus* isolates.

*Bacillus* Strains	Survival Rate (%)		
	pH 2	pH 3	Bile Salt 0.3%
K2.1	28.3 ± 1.16 ^def^	54.7 ± 1.00 ^e^	90.4 ± 1.53 ^bc^
K6.1	30.6 ± 3.00 ^de^	56.7 ± 0.58 ^e^	93.3 ± 2.31 ^abc^
K7.1	28.9 ± 3.51 ^def^	44.8 ± 3.61 ^gh^	94.0 ± 1.00 ^ab^
K9.1	25.5 ± 2.43 ^efg^	41.5 ± 1.59 ^h^	92.4 ± 2.31 ^abc^
K10.3	20.9 ± 0.98 ^fg^	53.8 ± 2.11 ^ef^	88.1 ± 3.94 ^bc^
K10.4	24.3 ± 3.07 ^efg^	56.7 ± 3.44 ^e^	86.1 ± 2.71 ^c^
K13.3	31.6 ± 1.57 ^de^	60.3 ± 2.57 ^de^	90.4 ± 1.97 ^bc^
K14.4	18.8 ± 2.49 ^g^	46.7 ± 3.05 ^gh^	87.3 ± 3.42 ^bc^
K15.2	40.4 ± 2.27 ^c^	65.2 ± 2.83 ^cde^	94.2 ± 2.45 ^ab^
K15.4	64.7 ± 4.73 ^ab^	72.7 ± 1.16 ^b^	99.0 ± 0.58 ^a^
K16.4	29.5 ± 4.73 ^de^	52.1 ± 2.43 ^f^	90.2 ± 2.31 ^bc^
K18.3	25.9 ± 3.22 ^efg^	40.6 ± 1.89 ^h^	85.9 ± 2.73 ^c^
K19.1	30.0 ± 2.46 ^de^	44.0 ± 2.01 ^gh^	88.8 ± 2.06 ^bc^
K21.3	36.1 ± 1.77 ^cd^	50.5 ± 1.39 ^fg^	89.6 ± 3.03 ^bc^
K22.6	64.3 ± 1.00 ^b^	71.4 ± 2.08 ^bc^	90.3 ± 3.05 ^bc^
K27.2	31.1 ± 2.88 ^de^	52.5 ± 4.01 ^f^	91.4 ± 3.95 ^abc^
K29.2	72.9 ± 2.52 ^a^	80.9 ± 2.00 ^a^	98.4 ± 0.58 ^a^

Note: Means in columns with different superscripts are statistically different at *p* < 0.05.

**Table 3:** The 16S rDNA identification results of the individual *Bacillus* species levels.

*Bacillus* Strains	Closest Species	Similarity (%)	Nucleotide (bp)	Accession Number
K2.1	*B. tequilensis* KCTC 13622	99.93	1449	OR534569
K6.1	*B. tequilensis* KCTC 13622	99.93	1449	OR534570
K7.1	*B. tequilensis* KCTC 13622	100.00	1450	OR534571
K15.4	*B. tequilensis* KCTC 13622	99.59	1460	OR534572
K22.6	*B. tequilensis* KCTC 13622	99.93	1450	OR534573
K29.2	*B. siamensis* KCTC 13613	99.93	1459	OR534574

**Table 4:** The *Bacillus* strains antibiotics susceptibility zone of inhibitions.

Antibiotics in 10 µg/mL	Antibiotics Inhibitory Zone Diameter (mm)					
	K2.1	K6.1	K7.1	K15.4	K22.6	K29.2
Erythromycin	31.5 ± 2.10 ^b^	34.5 ± 0.70 ^a^	30.5 ± 0.70 ^b^	32.0 ± 1.40 ^b^	29.5 ± 0.70 ^b^	31.0 ± 1.40 ^b^
Chloramphenicol	33.5 ± 0.70 ^a^	35.0 ± 1.40 ^a^	29.0 ± 1.00 ^b^	26.5 ± 0.70 ^c^	30.3 ± 1.00 ^b^	29.8 ± 0.30 ^b^
Streptomycin	17.3 ± 1.10 ^b^	9.3 ± 0.40 ^c^	10.5 ± 0.70 ^c^	8.3 ± 1.10 ^c^	15.0 ± 3.00 ^b^	22.8 ± 1.10 ^a^
Kanamycin	24.5 ± 0.70 ^a^	25.8 ± 0.40 ^a^	11.5 ± 0.07 ^d^	14.3 ± 0.40 ^c^	22.9 ± 1.20 ^b^	25.8 ± 0.40 ^a^
Ampicillin	29.0 ± 1.40 ^b^	37.0 ± 1.40 ^a^	25.5 ± 0.70 ^c^	9.5 ± 2.10 ^d^	30.5 ± 0.70 ^b^	30.0 ± 1.40 ^b^

Note: Means in columns with different superscripts are statistically different at *p* < 0.05.

## Data Availability

Data are contained within the article.
